# [μ-Bis(diphenyl­arsino)methane-1:2κ^2^ *As*:*As*′]nona­carbonyl-1κ^3^ *C*,2κ^3^ *C*,3κ^3^ *C*-[triphenyl­stibine-3κ*Sb*]-*triangulo*-triruthenium(0)

**DOI:** 10.1107/S1600536809049927

**Published:** 2009-12-24

**Authors:** Omar bin Shawkataly, Imthyaz Ahmed Khan, Chin Sing Yeap, Hoong-Kun Fun

**Affiliations:** a Chemical Sciences Programme, School of Distance Education, Universiti Sains Malaysia, 11800 USM, Penang, Malaysia; b X-ray Crystallography Unit, School of Physics, Universiti Sains Malaysia, 11800 USM, Penang, Malaysia

## Abstract

In the title *triangulo*-triruthenium compound, [Ru_3_(C_25_H_22_As_2_)(C_18_H_15_Sb)(CO)_9_], the bis­(diphenyl­arsino)methane ligand bridges an Ru—Ru bond and the monodentate stibine ligand bonds to the third Ru atom. Both the stibine and arsine ligands are equatorial with respect to the Ru_3_ triangle. Additionally, each Ru atom carries one equatorial and two axial terminal carbonyl ligands. The three stibine-substituted phenyl rings make dihedral angles of 84.3 (3), 80.4 (3) and 70.5 (3)° with each other. The dihedral angles between the two phenyl rings are 85.9 (3) and 75.2 (3)° for the two diphenyl­arsine groups. In the crystal packing, mol­ecules are linked into chains down the *c* axis *via* inter­molecular C—H⋯O hydrogen bonds. Weak inter­molecular C—H⋯π inter­actions further stabilize the crystal structure.

## Related literature

For general background to *triangulo*-triruthenium derivatives, see: Bruce *et al.* (1985 ▶, 1988*a* ▶,*b* ▶); Shawkataly *et al.* (1998 ▶, 2004 ▶, 2009 ▶). For related structures, see: Shawkataly *et al.* (2009 ▶). For the synthesis of μ-bis­(diphenylarsino)methanedecacarbonyl­triruthenium(0), see: Bruce *et al.* (1983 ▶). For the stability of the temperature controller used for the data collection, see: Cosier & Glazer (1986 ▶).
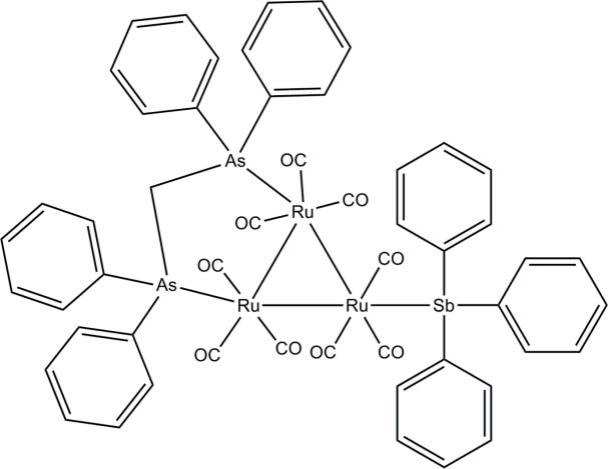


## Experimental

### 

#### Crystal data

[Ru_3_(C_25_H_22_As_2_)(C_18_H_15_Sb)(CO)_9_]
*M* *_r_* = 1380.62
Monoclinic, 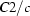

*a* = 42.3464 (6) Å
*b* = 11.6246 (2) Å
*c* = 20.1185 (3) Å
β = 91.823 (1)°
*V* = 9898.5 (3) Å^3^
*Z* = 8
Mo *K*α radiation
μ = 2.82 mm^−1^
*T* = 100 K
0.36 × 0.15 × 0.09 mm

#### Data collection

Bruker SMART APEXII CCD area-detector diffractometer
Absorption correction: multi-scan (**SADABS**; Bruker, 2005 ▶) *T*_min_ = 0.428, *T*_max_ = 0.796
55374 measured reflections
11349 independent reflections
8669 reflections with *I* > 2σ(*I*)
*R*_int_ = 0.042

#### Refinement

*R*[*F* ^2^ > 2σ(*F* ^2^)] = 0.037
*wR*(*F* ^2^) = 0.096
*S* = 1.08
11349 reflections
605 parameters
H-atom parameters constrained
Δρ_max_ = 1.45 e Å^−3^
Δρ_min_ = −1.24 e Å^−3^

### 

Data collection: *APEX2* (Bruker, 2005 ▶); cell refinement: *SAINT* (Bruker, 2005 ▶); data reduction: *SAINT*; program(s) used to solve structure: *SHELXTL* (Sheldrick, 2008 ▶); program(s) used to refine structure: *SHELXTL*; molecular graphics: *SHELXTL*; software used to prepare material for publication: *SHELXTL* and *PLATON* (Spek, 2009 ▶).

## Supplementary Material

Crystal structure: contains datablocks global, I. DOI: 10.1107/S1600536809049927/sj2682sup1.cif

Structure factors: contains datablocks I. DOI: 10.1107/S1600536809049927/sj2682Isup2.hkl

Additional supplementary materials:  crystallographic information; 3D view; checkCIF report

## Figures and Tables

**Table 1:** Hydrogen-bond geometry (Å, °)

*D*—H⋯*A*	*D*—H	H⋯*A*	*D*⋯*A*	*D*—H⋯*A*
C13—H13*B*⋯O8^i^	0.97	2.59	3.290 (7)	129
C23—H23*A*⋯*Cg*1^ii^	0.93	2.88	3.686 (6)	146
C34—H34*A*⋯*Cg*2^iii^	0.93	2.72	3.564 (6)	151

Symmetry codes: (i) 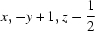; (ii) 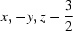; (iii) 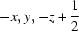. *Cg*1 and *Cg*2 are the centroids of the C14–C19 and C26–C31phenyl rings, respectively.

**Fig. 1.**
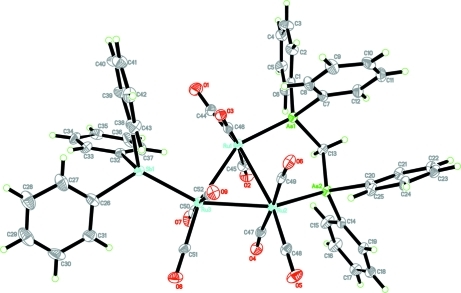
The molecular structure of the title compound with 30% probability ellipsoids for non-H atoms.

**Fig. 2.**
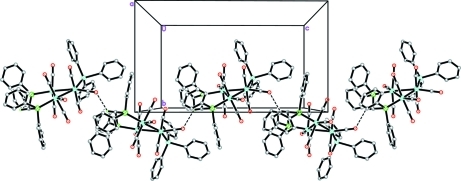
The crystal packing of the title compound, viewed down the a axis, showing the molecules linked along the c axis. Hydrogen atoms that are not involved in the hydrogen-bonding (dashed lines) have been omitted for clarity.

## supplementary crystallographic information

### Comment

*Triangulo*-triruthenium clusters are known for their interesting structural variations and related catalytic activity. A large number of substituted derivatives, Ru_3_(CO)_12-n_*L*_n_ (*L*= group 15 ligand) have been reported (Bruce *et al.*, 1985, 1988a,b). As part of our study on the substitution of transition metal-carbonyl clusters with mixed-ligand complexes, we have published several structures of *triangulo*-triruthenium-carbonyl clusters containing mixed P/As and P/Sb ligands (Shawkataly *et al.*, 1998, 2004, 2009). Herein we report the synthesis and structure of Ru_3_(C_18_H_15_Sb)(C_25_H_22_As_2_)(CO)_9_.

The bond lengths and angles of title compound (Fig. 1) are comparable to those found in a related structure (Shawkataly *et al.*, 2009). The bis(diphenylarsino)methane ligand bridges the Ru1—Ru2 bond and the monodentate stibine ligand bonds to the Ru3 atom. Both the stibine and arsine ligands are equatorial with respect to the Ru_3_ triangle. Additionally, each Ru atom carries one equatorial and two axial terminal carbonyl ligands. The three stibine substituted phenyl rings make dihedral angles (C26–C31/C32–C37, C26–C31/C38–C43 and C32–C37/C38–C43) of 84.3 (3), 80.4 (3) and 70.5 (3)° with each other respectively. The dihedral angles between the two phenyl rings (C1–C6/C7–C12 and C14–C19/C20–C25) are 85.9 (3) and 75.2 (3)° for the two diphenylarsino groups respectively.

In the crystal packing (Fig. 2), the molecules are linked together into chains *via* intermolecular C13—H13B···O8 along *c* axis. Weak intermolecular C—H···π interactions further stabilize the crystal structure (Table 1).

### Experimental

All manipulations were performed under a dry oxygen-free dinitrogen atmosphere using standard Schlenk techniques, all solvents were dried over sodium and distilled from sodium benzophenone ketyl under nitrogen. Triphenylstibine (Fluka) used as received and µ-bis(diphenylarsino)methanedecacarbonyltriruthenium(0) (Bruce *et al.*, 1983) was prepared by reported procedure. The title compound was obtained by refluxing equimolar quantities of Ru_3_(CO)_10_(µ-Ph_2_AsCH_2_AsPh_2_) (105.5 mg, 0.1 mmol) and triphenylstibine (35.3 mg, 0.1 mmol) in hexane under nitrogen atmosphere. Crystals suitable for X-ray diffraction were grown by slow solvent / solvent diffusion of CH_3_OH into CH_2_Cl_2_.

### Refinement

All hydrogen atoms were positioned geometrically and refined using a riding model with C—H = 0.93–0.97 Å and *U*_iso_(H) = 1.2 *U*_eq_(C). The final difference Fourier map reveals high peaks, ~1.5 e Å^-3^, two of which are quite separate from the heavy atoms. These could be due to the presence of additional solvent, possibly methanol, and perhaps at partial occupancy. Attempts to produce a satisfactory model of this solvent were not successful.

### Crystal data

**Table:**

[Ru_3_(C_25_H_22_As_2_)(C_18_H_15_Sb)(CO)_9_]	*F*(000) = 5360
*M**_r_* = 1380.62	*D*_x_ = 1.853 Mg m^−^^3^
Monoclinic, *C*2/*c*	Mo *K*α radiation, λ = 0.71073 Å
Hall symbol: -C 2yc	Cell parameters from 9674 reflections
*a* = 42.3464 (6) Å	θ = 2.3–30.6°
*b* = 11.6246 (2) Å	µ = 2.82 mm^−^^1^
*c* = 20.1185 (3) Å	*T* = 100 K
β = 91.823 (1)°	Block, red
*V* = 9898.5 (3) Å^3^	0.36 × 0.15 × 0.09 mm
*Z* = 8	

### Data collection

**Table:**

Bruker SMART APEXII CCD area-detector diffractometer	11349 independent reflections
Radiation source: fine-focus sealed tube	8669 reflections with *I* > 2σ(*I*)
graphite	*R*_int_ = 0.042
φ and ω scans	θ_max_ = 27.5°, θ_min_ = 2.0°
Absorption correction: multi-scan (*SADABS*; Bruker, 2005)	*h* = −54→50
*T*_min_ = 0.428, *T*_max_ = 0.796	*k* = −14→15
55374 measured reflections	*l* = −25→26

### Refinement

**Table:**

Refinement on *F*^2^	Primary atom site location: structure-invariant direct methods
Least-squares matrix: full	Secondary atom site location: difference Fourier map
*R*[*F*^2^ > 2σ(*F*^2^)] = 0.037	Hydrogen site location: inferred from neighbouring sites
*wR*(*F*^2^) = 0.096	H-atom parameters constrained
*S* = 1.08	*w* = 1/[σ^2^(*F*_o_^2^) + (0.027*P*)^2^ + 103.4772*P*] where *P* = (*F*_o_^2^ + 2*F*_c_^2^)/3
11349 reflections	(Δ/σ)_max_ = 0.001
605 parameters	Δρ_max_ = 1.45 e Å^−^^3^
0 restraints	Δρ_min_ = −1.24 e Å^−^^3^

### Special details

**Table:**

Experimental. The crystal was placed in the cold stream of an Oxford Cyrosystems Cobra open-flow nitrogen cryostat (Cosier & Glazer, 1986) operating at 100.0 (1) K.
Geometry. All e.s.d.'s (except the e.s.d. in the dihedral angle between two l.s. planes) are estimated using the full covariance matrix. The cell e.s.d.'s are taken into account individually in the estimation of e.s.d.'s in distances, angles and torsion angles; correlations between e.s.d.'s in cell parameters are only used when they are defined by crystal symmetry. An approximate (isotropic) treatment of cell e.s.d.'s is used for estimating e.s.d.'s involving l.s. planes.
Refinement. Refinement of *F*^2^ against ALL reflections. The weighted *R*-factor *wR* and goodness of fit *S* are based on *F*^2^, conventional *R*-factors *R* are based on *F*, with *F* set to zero for negative *F*^2^. The threshold expression of *F*^2^ > σ(*F*^2^) is used only for calculating *R*-factors(gt) *etc*. and is not relevant to the choice of reflections for refinement. *R*-factors based on *F*^2^ are statistically about twice as large as those based on *F*, and *R*- factors based on ALL data will be even larger.

### Fractional atomic coordinates and isotropic or equivalent isotropic displacement parameters (Å^2^)

**Table:**

	*x*	*y*	*z*	*U*_iso_*/*U*_eq_	
Sb1	0.084142 (8)	0.82926 (3)	0.122293 (17)	0.02485 (8)	
Ru1	0.111861 (9)	0.69580 (3)	−0.05368 (2)	0.02414 (10)	
Ru2	0.173966 (9)	0.62369 (3)	−0.01095 (2)	0.02287 (9)	
Ru3	0.132574 (9)	0.71467 (3)	0.08382 (2)	0.02524 (10)	
As1	0.124706 (12)	0.65958 (4)	−0.16939 (3)	0.02403 (11)	
As2	0.185533 (11)	0.52317 (4)	−0.11339 (3)	0.02262 (11)	
O1	0.04220 (10)	0.7484 (5)	−0.0798 (2)	0.0569 (13)	
O2	0.09819 (10)	0.4372 (3)	−0.0433 (2)	0.0431 (10)	
O3	0.12504 (9)	0.9554 (3)	−0.04611 (18)	0.0311 (8)	
O4	0.15850 (10)	0.3916 (3)	0.0537 (2)	0.0409 (10)	
O5	0.23787 (10)	0.6078 (4)	0.0599 (2)	0.0491 (11)	
O6	0.19089 (9)	0.8586 (3)	−0.0700 (2)	0.0355 (9)	
O7	0.09752 (10)	0.4866 (3)	0.1062 (2)	0.0467 (11)	
O8	0.16762 (9)	0.6817 (4)	0.2157 (2)	0.0432 (10)	
O9	0.17354 (9)	0.9301 (3)	0.06805 (19)	0.0338 (9)	
C1	0.08858 (12)	0.6283 (4)	−0.2287 (3)	0.0268 (11)	
C2	0.07734 (14)	0.7137 (5)	−0.2713 (3)	0.0382 (13)	
H2A	0.0887	0.7816	−0.2753	0.046*	
C3	0.04930 (15)	0.6994 (5)	−0.3082 (3)	0.0422 (15)	
H3A	0.0416	0.7582	−0.3356	0.051*	
C4	0.03303 (14)	0.5975 (6)	−0.3038 (3)	0.0388 (14)	
H4A	0.0143	0.5876	−0.3284	0.047*	
C5	0.04410 (13)	0.5110 (5)	−0.2639 (3)	0.0398 (14)	
H5A	0.0332	0.4417	−0.2622	0.048*	
C6	0.07193 (13)	0.5261 (5)	−0.2253 (3)	0.0341 (13)	
H6A	0.0792	0.4673	−0.1975	0.041*	
C7	0.14865 (12)	0.7655 (5)	−0.2226 (3)	0.0279 (11)	
C8	0.14999 (14)	0.8819 (5)	−0.2059 (3)	0.0351 (13)	
H8A	0.1393	0.9089	−0.1693	0.042*	
C9	0.16728 (15)	0.9577 (5)	−0.2442 (3)	0.0400 (14)	
H9A	0.1679	1.0354	−0.2334	0.048*	
C10	0.18329 (14)	0.9184 (5)	−0.2973 (3)	0.0374 (14)	
H10A	0.1957	0.9689	−0.3212	0.045*	
C11	0.18129 (15)	0.8051 (6)	−0.3158 (3)	0.0441 (15)	
H11A	0.1914	0.7796	−0.3534	0.053*	
C12	0.16402 (14)	0.7281 (5)	−0.2780 (3)	0.0367 (13)	
H12A	0.1629	0.6511	−0.2903	0.044*	
C13	0.14903 (12)	0.5166 (4)	−0.1756 (3)	0.0268 (11)	
H13A	0.1358	0.4515	−0.1649	0.032*	
H13B	0.1561	0.5068	−0.2206	0.032*	
C14	0.19684 (12)	0.3618 (4)	−0.1015 (3)	0.0259 (11)	
C15	0.17403 (14)	0.2789 (5)	−0.0939 (4)	0.0428 (15)	
H15A	0.1528	0.2983	−0.0989	0.051*	
C16	0.18254 (15)	0.1664 (5)	−0.0788 (4)	0.0462 (16)	
H16A	0.1670	0.1107	−0.0747	0.055*	
C17	0.21371 (15)	0.1370 (5)	−0.0699 (3)	0.0377 (13)	
H17A	0.2193	0.0617	−0.0591	0.045*	
C18	0.23675 (14)	0.2198 (5)	−0.0770 (3)	0.0353 (13)	
H18A	0.2579	0.2003	−0.0711	0.042*	
C19	0.22837 (12)	0.3321 (4)	−0.0930 (3)	0.0297 (11)	
H19A	0.2440	0.3874	−0.0980	0.036*	
C20	0.21906 (11)	0.5759 (4)	−0.1697 (3)	0.0238 (10)	
C21	0.22437 (12)	0.5219 (5)	−0.2302 (3)	0.0311 (12)	
H21A	0.2123	0.4584	−0.2431	0.037*	
C22	0.24741 (12)	0.5623 (5)	−0.2708 (3)	0.0317 (12)	
H22A	0.2509	0.5258	−0.3110	0.038*	
C23	0.26547 (12)	0.6576 (5)	−0.2520 (3)	0.0324 (12)	
H23A	0.2807	0.6860	−0.2800	0.039*	
C24	0.26066 (12)	0.7096 (5)	−0.1917 (3)	0.0327 (12)	
H24A	0.2730	0.7724	−0.1786	0.039*	
C25	0.23753 (12)	0.6687 (4)	−0.1502 (3)	0.0285 (11)	
H25A	0.2345	0.7039	−0.1094	0.034*	
C26	0.08319 (14)	0.8791 (5)	0.2245 (3)	0.0362 (13)	
C27	0.06664 (18)	0.9775 (6)	0.2432 (3)	0.0527 (18)	
H27A	0.0580	1.0250	0.2101	0.063*	
C28	0.0626 (2)	1.0070 (6)	0.3096 (4)	0.062 (2)	
H28A	0.0513	1.0722	0.3209	0.074*	
C29	0.07591 (19)	0.9367 (8)	0.3582 (4)	0.062 (2)	
H29A	0.0732	0.9541	0.4027	0.074*	
C30	0.09304 (15)	0.8420 (8)	0.3418 (3)	0.060 (2)	
H30A	0.1025	0.7974	0.3752	0.072*	
C31	0.09648 (14)	0.8112 (7)	0.2746 (3)	0.0491 (17)	
H31A	0.1077	0.7453	0.2639	0.059*	
C32	0.03824 (12)	0.7518 (4)	0.1172 (3)	0.0266 (11)	
C33	0.01478 (13)	0.7924 (5)	0.1571 (3)	0.0355 (13)	
H33A	0.0188	0.8555	0.1845	0.043*	
C34	−0.01436 (13)	0.7407 (5)	0.1569 (3)	0.0391 (14)	
H34A	−0.0299	0.7678	0.1846	0.047*	
C35	−0.02059 (13)	0.6480 (6)	0.1154 (3)	0.0424 (15)	
H35A	−0.0406	0.6146	0.1139	0.051*	
C36	0.00261 (16)	0.6057 (6)	0.0767 (3)	0.0480 (17)	
H36A	−0.0014	0.5413	0.0503	0.058*	
C37	0.03201 (13)	0.6578 (5)	0.0764 (3)	0.0350 (13)	
H37A	0.0475	0.6299	0.0489	0.042*	
C38	0.07462 (13)	0.9943 (4)	0.0789 (3)	0.0278 (11)	
C39	0.04575 (14)	1.0187 (5)	0.0475 (3)	0.0376 (14)	
H39A	0.0301	0.9624	0.0443	0.045*	
C40	0.04024 (16)	1.1274 (5)	0.0207 (3)	0.0445 (15)	
H40A	0.0208	1.1443	0.0001	0.053*	
C41	0.06361 (15)	1.2100 (5)	0.0246 (3)	0.0416 (14)	
H41A	0.0601	1.2821	0.0058	0.050*	
C42	0.09210 (14)	1.1861 (5)	0.0562 (3)	0.0377 (14)	
H42A	0.1077	1.2423	0.0591	0.045*	
C43	0.09760 (13)	1.0786 (5)	0.0837 (3)	0.0328 (12)	
H43A	0.1168	1.0629	0.1055	0.039*	
C44	0.06855 (14)	0.7302 (5)	−0.0681 (3)	0.0366 (13)	
C45	0.10430 (13)	0.5344 (5)	−0.0440 (3)	0.0335 (12)	
C46	0.12143 (12)	0.8580 (5)	−0.0476 (3)	0.0281 (11)	
C47	0.16233 (13)	0.4801 (5)	0.0298 (3)	0.0313 (12)	
C48	0.21364 (13)	0.6139 (5)	0.0330 (3)	0.0320 (12)	
C49	0.18295 (12)	0.7728 (4)	−0.0483 (3)	0.0274 (11)	
C50	0.11020 (13)	0.5703 (5)	0.0947 (3)	0.0360 (13)	
C51	0.15435 (12)	0.6939 (5)	0.1651 (3)	0.0312 (12)	
C52	0.15780 (13)	0.8488 (5)	0.0705 (3)	0.0290 (11)	

### Atomic displacement parameters (Å^2^)

**Table:**

	*U*^11^	*U*^22^	*U*^33^	*U*^12^	*U*^13^	*U*^23^
Sb1	0.02101 (17)	0.02523 (17)	0.02838 (18)	−0.00263 (13)	0.00167 (13)	0.00197 (14)
Ru1	0.0184 (2)	0.0245 (2)	0.0297 (2)	−0.00163 (15)	0.00413 (16)	0.00320 (16)
Ru2	0.01783 (19)	0.02012 (19)	0.0308 (2)	−0.00182 (15)	0.00339 (16)	−0.00040 (16)
Ru3	0.0209 (2)	0.0246 (2)	0.0304 (2)	−0.00150 (16)	0.00461 (16)	0.00228 (17)
As1	0.0213 (3)	0.0211 (2)	0.0299 (3)	−0.00367 (19)	0.0037 (2)	0.0000 (2)
As2	0.0189 (2)	0.0165 (2)	0.0326 (3)	−0.00326 (18)	0.0048 (2)	−0.0012 (2)
O1	0.027 (2)	0.087 (4)	0.057 (3)	0.008 (2)	0.003 (2)	0.015 (3)
O2	0.043 (2)	0.032 (2)	0.054 (3)	−0.0134 (18)	−0.001 (2)	0.0059 (19)
O3	0.038 (2)	0.0244 (19)	0.031 (2)	0.0034 (16)	0.0047 (17)	0.0020 (15)
O4	0.042 (2)	0.030 (2)	0.052 (3)	−0.0002 (18)	0.011 (2)	0.0113 (19)
O5	0.033 (2)	0.046 (3)	0.068 (3)	0.0054 (19)	−0.014 (2)	−0.013 (2)
O6	0.028 (2)	0.0215 (19)	0.057 (3)	−0.0020 (15)	0.0103 (18)	0.0024 (17)
O7	0.051 (3)	0.027 (2)	0.064 (3)	−0.0077 (19)	0.024 (2)	0.002 (2)
O8	0.031 (2)	0.053 (3)	0.046 (3)	−0.0016 (19)	−0.0013 (19)	0.020 (2)
O9	0.039 (2)	0.0269 (19)	0.035 (2)	−0.0100 (17)	0.0042 (17)	−0.0029 (16)
C1	0.023 (3)	0.027 (3)	0.031 (3)	−0.003 (2)	0.006 (2)	−0.004 (2)
C2	0.041 (3)	0.032 (3)	0.041 (3)	−0.008 (3)	0.000 (3)	0.001 (3)
C3	0.041 (3)	0.042 (3)	0.043 (4)	−0.010 (3)	−0.010 (3)	0.009 (3)
C4	0.029 (3)	0.054 (4)	0.033 (3)	−0.008 (3)	−0.004 (2)	−0.002 (3)
C5	0.031 (3)	0.037 (3)	0.051 (4)	−0.012 (2)	0.000 (3)	0.001 (3)
C6	0.030 (3)	0.032 (3)	0.041 (3)	−0.009 (2)	0.002 (2)	0.005 (2)
C7	0.024 (3)	0.030 (3)	0.030 (3)	−0.005 (2)	0.003 (2)	0.002 (2)
C8	0.046 (3)	0.030 (3)	0.031 (3)	−0.009 (2)	0.011 (3)	−0.004 (2)
C9	0.049 (4)	0.031 (3)	0.041 (3)	−0.016 (3)	0.006 (3)	−0.001 (3)
C10	0.037 (3)	0.042 (3)	0.033 (3)	−0.014 (3)	0.000 (3)	0.012 (3)
C11	0.044 (4)	0.047 (4)	0.042 (4)	0.006 (3)	0.019 (3)	0.006 (3)
C12	0.041 (3)	0.032 (3)	0.038 (3)	0.000 (2)	0.010 (3)	−0.001 (2)
C13	0.024 (3)	0.020 (2)	0.036 (3)	−0.0026 (19)	0.000 (2)	−0.005 (2)
C14	0.028 (3)	0.020 (2)	0.030 (3)	−0.002 (2)	0.006 (2)	−0.002 (2)
C15	0.029 (3)	0.024 (3)	0.076 (5)	−0.002 (2)	0.002 (3)	0.002 (3)
C16	0.041 (4)	0.023 (3)	0.075 (5)	−0.012 (3)	0.002 (3)	0.003 (3)
C17	0.051 (4)	0.021 (3)	0.041 (3)	0.001 (2)	0.002 (3)	−0.002 (2)
C18	0.036 (3)	0.032 (3)	0.037 (3)	0.006 (2)	0.003 (3)	0.001 (2)
C19	0.028 (3)	0.023 (2)	0.039 (3)	−0.002 (2)	0.006 (2)	0.000 (2)
C20	0.019 (2)	0.016 (2)	0.036 (3)	−0.0029 (18)	0.003 (2)	0.002 (2)
C21	0.027 (3)	0.026 (3)	0.040 (3)	−0.005 (2)	0.003 (2)	−0.003 (2)
C22	0.027 (3)	0.035 (3)	0.034 (3)	0.001 (2)	0.005 (2)	−0.001 (2)
C23	0.024 (3)	0.027 (3)	0.046 (3)	−0.001 (2)	0.008 (2)	0.005 (2)
C24	0.024 (3)	0.027 (3)	0.048 (3)	−0.004 (2)	0.010 (2)	−0.001 (2)
C25	0.023 (3)	0.023 (2)	0.040 (3)	−0.001 (2)	0.005 (2)	−0.004 (2)
C26	0.036 (3)	0.045 (3)	0.028 (3)	−0.017 (3)	0.000 (2)	0.004 (3)
C27	0.081 (5)	0.040 (4)	0.038 (4)	−0.012 (3)	0.011 (3)	−0.005 (3)
C28	0.100 (6)	0.042 (4)	0.044 (4)	−0.016 (4)	0.014 (4)	−0.009 (3)
C29	0.061 (5)	0.086 (6)	0.038 (4)	−0.034 (5)	0.001 (3)	−0.013 (4)
C30	0.025 (3)	0.116 (7)	0.037 (4)	−0.010 (4)	−0.004 (3)	0.026 (4)
C31	0.022 (3)	0.081 (5)	0.044 (4)	0.002 (3)	0.001 (3)	0.010 (3)
C32	0.027 (3)	0.024 (2)	0.030 (3)	−0.003 (2)	0.001 (2)	0.010 (2)
C33	0.027 (3)	0.032 (3)	0.048 (4)	0.004 (2)	0.001 (3)	0.001 (3)
C34	0.025 (3)	0.041 (3)	0.052 (4)	0.006 (2)	0.008 (3)	0.005 (3)
C35	0.021 (3)	0.061 (4)	0.044 (4)	−0.013 (3)	−0.005 (3)	0.017 (3)
C36	0.049 (4)	0.062 (4)	0.033 (3)	−0.026 (3)	0.003 (3)	−0.008 (3)
C37	0.032 (3)	0.041 (3)	0.033 (3)	−0.007 (2)	0.010 (2)	−0.004 (2)
C38	0.032 (3)	0.025 (3)	0.027 (3)	−0.002 (2)	0.005 (2)	0.002 (2)
C39	0.042 (3)	0.034 (3)	0.037 (3)	−0.015 (3)	−0.008 (3)	0.010 (2)
C40	0.047 (4)	0.041 (3)	0.044 (4)	−0.008 (3)	−0.015 (3)	0.014 (3)
C41	0.053 (4)	0.031 (3)	0.041 (3)	−0.007 (3)	0.000 (3)	0.007 (3)
C42	0.039 (3)	0.029 (3)	0.046 (4)	−0.012 (2)	0.009 (3)	−0.002 (3)
C43	0.025 (3)	0.026 (3)	0.047 (3)	−0.002 (2)	0.003 (2)	−0.003 (2)
C44	0.029 (3)	0.045 (3)	0.037 (3)	0.003 (3)	0.007 (2)	0.006 (3)
C45	0.025 (3)	0.039 (3)	0.036 (3)	−0.009 (2)	0.000 (2)	0.002 (2)
C46	0.023 (3)	0.035 (3)	0.026 (3)	0.001 (2)	0.003 (2)	0.000 (2)
C47	0.030 (3)	0.027 (3)	0.037 (3)	0.002 (2)	0.004 (2)	0.001 (2)
C48	0.030 (3)	0.027 (3)	0.039 (3)	0.001 (2)	0.000 (2)	−0.008 (2)
C49	0.018 (2)	0.028 (3)	0.036 (3)	0.000 (2)	0.006 (2)	−0.003 (2)
C50	0.031 (3)	0.030 (3)	0.048 (4)	0.003 (2)	0.014 (3)	0.000 (3)
C51	0.020 (3)	0.036 (3)	0.038 (3)	0.003 (2)	0.003 (2)	0.013 (2)
C52	0.032 (3)	0.030 (3)	0.025 (3)	0.010 (2)	−0.001 (2)	−0.003 (2)

### Geometric parameters (Å, °)

**Table:**

Sb1—C26	2.138 (6)	C13—H13B	0.9700
Sb1—C38	2.141 (5)	C14—C15	1.377 (7)
Sb1—C32	2.142 (5)	C14—C19	1.385 (7)
Sb1—Ru3	2.5847 (5)	C15—C16	1.387 (8)
Ru1—C44	1.891 (6)	C15—H15A	0.9300
Ru1—C45	1.914 (6)	C16—C17	1.370 (9)
Ru1—C46	1.932 (6)	C16—H16A	0.9300
Ru1—As1	2.4439 (7)	C17—C18	1.382 (8)
Ru1—Ru2	2.8661 (6)	C17—H17A	0.9300
Ru1—Ru3	2.8838 (6)	C18—C19	1.387 (7)
Ru2—C48	1.876 (6)	C18—H18A	0.9300
Ru2—C47	1.930 (5)	C19—H19A	0.9300
Ru2—C49	1.932 (5)	C20—C25	1.382 (7)
Ru2—As2	2.4325 (6)	C20—C21	1.394 (7)
Ru2—Ru3	2.8354 (6)	C21—C22	1.375 (7)
Ru3—C51	1.866 (6)	C21—H21A	0.9300
Ru3—C52	1.914 (6)	C22—C23	1.392 (7)
Ru3—C50	1.942 (6)	C22—H22A	0.9300
As1—C7	1.939 (5)	C23—C24	1.377 (8)
As1—C1	1.944 (5)	C23—H23A	0.9300
As1—C13	1.961 (5)	C24—C25	1.390 (7)
As2—C20	1.943 (5)	C24—H24A	0.9300
As2—C14	1.948 (5)	C25—H25A	0.9300
As2—C13	1.959 (5)	C26—C31	1.385 (9)
O1—C44	1.153 (7)	C26—C27	1.399 (9)
O2—C45	1.160 (7)	C27—C28	1.396 (9)
O3—C46	1.143 (6)	C27—H27A	0.9300
O4—C47	1.150 (6)	C28—C29	1.380 (11)
O5—C48	1.148 (6)	C28—H28A	0.9300
O6—C49	1.143 (6)	C29—C30	1.364 (11)
O7—C50	1.139 (7)	C29—H29A	0.9300
O8—C51	1.156 (7)	C30—C31	1.411 (10)
O9—C52	1.158 (6)	C30—H30A	0.9300
C1—C6	1.385 (7)	C31—H31A	0.9300
C1—C2	1.386 (8)	C32—C33	1.380 (8)
C2—C3	1.391 (8)	C32—C37	1.387 (7)
C2—H2A	0.9300	C33—C34	1.373 (8)
C3—C4	1.374 (8)	C33—H33A	0.9300
C3—H3A	0.9300	C34—C35	1.382 (9)
C4—C5	1.361 (8)	C34—H34A	0.9300
C4—H4A	0.9300	C35—C36	1.364 (9)
C5—C6	1.401 (8)	C35—H35A	0.9300
C5—H5A	0.9300	C36—C37	1.385 (8)
C6—H6A	0.9300	C36—H36A	0.9300
C7—C12	1.379 (8)	C37—H37A	0.9300
C7—C8	1.395 (7)	C38—C43	1.382 (7)
C8—C9	1.393 (7)	C38—C39	1.388 (8)
C8—H8A	0.9300	C39—C40	1.391 (8)
C9—C10	1.362 (8)	C39—H39A	0.9300
C9—H9A	0.9300	C40—C41	1.380 (8)
C10—C11	1.371 (9)	C40—H40A	0.9300
C10—H10A	0.9300	C41—C42	1.374 (9)
C11—C12	1.396 (8)	C41—H41A	0.9300
C11—H11A	0.9300	C42—C43	1.384 (8)
C12—H12A	0.9300	C42—H42A	0.9300
C13—H13A	0.9700	C43—H43A	0.9300
			
C26—Sb1—C38	98.0 (2)	As2—C13—H13B	109.8
C26—Sb1—C32	96.6 (2)	As1—C13—H13B	109.8
C38—Sb1—C32	101.44 (19)	H13A—C13—H13B	108.2
C26—Sb1—Ru3	117.84 (17)	C15—C14—C19	119.2 (5)
C38—Sb1—Ru3	118.75 (14)	C15—C14—As2	121.2 (4)
C32—Sb1—Ru3	119.78 (14)	C19—C14—As2	119.2 (4)
C44—Ru1—C45	93.4 (2)	C14—C15—C16	120.4 (6)
C44—Ru1—C46	90.2 (2)	C14—C15—H15A	119.8
C45—Ru1—C46	170.3 (2)	C16—C15—H15A	119.8
C44—Ru1—As1	97.71 (18)	C17—C16—C15	120.5 (5)
C45—Ru1—As1	88.34 (17)	C17—C16—H16A	119.8
C46—Ru1—As1	100.15 (15)	C15—C16—H16A	119.8
C44—Ru1—Ru2	169.88 (17)	C16—C17—C18	119.5 (5)
C45—Ru1—Ru2	80.65 (16)	C16—C17—H17A	120.3
C46—Ru1—Ru2	94.46 (15)	C18—C17—H17A	120.3
As1—Ru1—Ru2	90.310 (19)	C17—C18—C19	120.2 (5)
C44—Ru1—Ru3	113.30 (18)	C17—C18—H18A	119.9
C45—Ru1—Ru3	91.36 (17)	C19—C18—H18A	119.9
C46—Ru1—Ru3	78.92 (16)	C14—C19—C18	120.1 (5)
As1—Ru1—Ru3	148.95 (2)	C14—C19—H19A	119.9
Ru2—Ru1—Ru3	59.090 (14)	C18—C19—H19A	119.9
C48—Ru2—C47	89.1 (2)	C25—C20—C21	119.6 (5)
C48—Ru2—C49	93.0 (2)	C25—C20—As2	119.9 (4)
C47—Ru2—C49	175.6 (2)	C21—C20—As2	120.5 (4)
C48—Ru2—As2	99.70 (16)	C22—C21—C20	120.2 (5)
C47—Ru2—As2	90.30 (17)	C22—C21—H21A	119.9
C49—Ru2—As2	93.09 (16)	C20—C21—H21A	119.9
C48—Ru2—Ru3	105.62 (17)	C21—C22—C23	120.3 (5)
C47—Ru2—Ru3	82.27 (16)	C21—C22—H22A	119.8
C49—Ru2—Ru3	93.47 (15)	C23—C22—H22A	119.9
As2—Ru2—Ru3	153.43 (2)	C24—C23—C22	119.5 (5)
C48—Ru2—Ru1	163.36 (16)	C24—C23—H23A	120.2
C47—Ru2—Ru1	97.82 (16)	C22—C23—H23A	120.2
C49—Ru2—Ru1	79.10 (15)	C23—C24—C25	120.4 (5)
As2—Ru2—Ru1	95.39 (2)	C23—C24—H24A	119.8
Ru3—Ru2—Ru1	60.766 (14)	C25—C24—H24A	119.8
C51—Ru3—C52	88.0 (2)	C20—C25—C24	120.0 (5)
C51—Ru3—C50	91.1 (2)	C20—C25—H25A	120.0
C52—Ru3—C50	174.8 (2)	C24—C25—H25A	120.0
C51—Ru3—Sb1	100.32 (17)	C31—C26—C27	117.8 (6)
C52—Ru3—Sb1	94.24 (15)	C31—C26—Sb1	121.7 (5)
C50—Ru3—Sb1	90.98 (16)	C27—C26—Sb1	120.3 (5)
C51—Ru3—Ru2	103.99 (17)	C28—C27—C26	122.3 (7)
C52—Ru3—Ru2	81.37 (15)	C28—C27—H27A	118.8
C50—Ru3—Ru2	93.86 (16)	C26—C27—H27A	118.8
Sb1—Ru3—Ru2	155.09 (2)	C29—C28—C27	118.3 (8)
C51—Ru3—Ru1	163.12 (17)	C29—C28—H28A	120.9
C52—Ru3—Ru1	94.69 (16)	C27—C28—H28A	120.9
C50—Ru3—Ru1	84.69 (19)	C30—C29—C28	121.0 (7)
Sb1—Ru3—Ru1	96.108 (17)	C30—C29—H29A	119.5
Ru2—Ru3—Ru1	60.144 (14)	C28—C29—H29A	119.5
C7—As1—C1	101.2 (2)	C29—C30—C31	120.6 (7)
C7—As1—C13	102.6 (2)	C29—C30—H30A	119.7
C1—As1—C13	102.0 (2)	C31—C30—H30A	119.7
C7—As1—Ru1	123.55 (16)	C26—C31—C30	120.0 (7)
C1—As1—Ru1	114.97 (15)	C26—C31—H31A	120.0
C13—As1—Ru1	109.85 (16)	C30—C31—H31A	120.0
C20—As2—C14	101.2 (2)	C33—C32—C37	119.1 (5)
C20—As2—C13	102.5 (2)	C33—C32—Sb1	119.8 (4)
C14—As2—C13	103.1 (2)	C37—C32—Sb1	121.0 (4)
C20—As2—Ru2	120.83 (15)	C34—C33—C32	120.8 (6)
C14—As2—Ru2	114.44 (15)	C34—C33—H33A	119.6
C13—As2—Ru2	112.55 (15)	C32—C33—H33A	119.6
C6—C1—C2	118.7 (5)	C33—C34—C35	119.8 (6)
C6—C1—As1	121.5 (4)	C33—C34—H34A	120.1
C2—C1—As1	119.6 (4)	C35—C34—H34A	120.1
C1—C2—C3	121.0 (5)	C36—C35—C34	119.9 (5)
C1—C2—H2A	119.5	C36—C35—H35A	120.1
C3—C2—H2A	119.5	C34—C35—H35A	120.1
C4—C3—C2	119.3 (6)	C35—C36—C37	120.6 (6)
C4—C3—H3A	120.3	C35—C36—H36A	119.7
C2—C3—H3A	120.3	C37—C36—H36A	119.7
C5—C4—C3	120.7 (5)	C36—C37—C32	119.7 (5)
C5—C4—H4A	119.6	C36—C37—H37A	120.1
C3—C4—H4A	119.6	C32—C37—H37A	120.1
C4—C5—C6	120.1 (5)	C43—C38—C39	119.7 (5)
C4—C5—H5A	119.9	C43—C38—Sb1	118.9 (4)
C6—C5—H5A	119.9	C39—C38—Sb1	121.3 (4)
C1—C6—C5	120.1 (5)	C38—C39—C40	119.8 (5)
C1—C6—H6A	120.0	C38—C39—H39A	120.1
C5—C6—H6A	120.0	C40—C39—H39A	120.1
C12—C7—C8	118.9 (5)	C41—C40—C39	119.9 (6)
C12—C7—As1	121.0 (4)	C41—C40—H40A	120.0
C8—C7—As1	120.1 (4)	C39—C40—H40A	120.0
C9—C8—C7	119.9 (5)	C42—C41—C40	120.3 (6)
C9—C8—H8A	120.0	C42—C41—H41A	119.9
C7—C8—H8A	120.0	C40—C41—H41A	119.9
C10—C9—C8	120.4 (5)	C41—C42—C43	120.1 (5)
C10—C9—H9A	119.8	C41—C42—H42A	120.0
C8—C9—H9A	119.8	C43—C42—H42A	120.0
C9—C10—C11	120.5 (5)	C38—C43—C42	120.2 (5)
C9—C10—H10A	119.8	C38—C43—H43A	119.9
C11—C10—H10A	119.8	C42—C43—H43A	119.9
C10—C11—C12	119.8 (6)	O1—C44—Ru1	176.7 (6)
C10—C11—H11A	120.1	O2—C45—Ru1	174.0 (5)
C12—C11—H11A	120.1	O3—C46—Ru1	175.1 (5)
C7—C12—C11	120.5 (5)	O4—C47—Ru2	173.2 (5)
C7—C12—H12A	119.8	O5—C48—Ru2	179.8 (6)
C11—C12—H12A	119.8	O6—C49—Ru2	174.2 (4)
As2—C13—As1	109.4 (2)	O7—C50—Ru3	174.7 (6)
As2—C13—H13A	109.8	O8—C51—Ru3	179.3 (5)
As1—C13—H13A	109.8	O9—C52—Ru3	174.4 (5)
			
C44—Ru1—Ru2—C48	−80.4 (13)	Ru1—Ru2—As2—C14	−123.40 (17)
C45—Ru1—Ru2—C48	−134.6 (6)	C48—Ru2—As2—C13	−179.1 (2)
C46—Ru1—Ru2—C48	36.9 (6)	C47—Ru2—As2—C13	91.8 (2)
As1—Ru1—Ru2—C48	137.1 (6)	C49—Ru2—As2—C13	−85.4 (2)
Ru3—Ru1—Ru2—C48	−37.4 (6)	Ru3—Ru2—As2—C13	18.65 (17)
C44—Ru1—Ru2—C47	33.5 (11)	Ru1—Ru2—As2—C13	−6.11 (16)
C45—Ru1—Ru2—C47	−20.7 (2)	C7—As1—C1—C6	153.1 (4)
C46—Ru1—Ru2—C47	150.8 (2)	C13—As1—C1—C6	47.5 (5)
As1—Ru1—Ru2—C47	−108.97 (17)	Ru1—As1—C1—C6	−71.3 (5)
Ru3—Ru1—Ru2—C47	76.56 (17)	C7—As1—C1—C2	−32.7 (5)
C44—Ru1—Ru2—C49	−143.3 (11)	C13—As1—C1—C2	−138.4 (4)
C45—Ru1—Ru2—C49	162.5 (2)	Ru1—As1—C1—C2	102.8 (4)
C46—Ru1—Ru2—C49	−26.0 (2)	C6—C1—C2—C3	2.4 (9)
As1—Ru1—Ru2—C49	74.19 (16)	As1—C1—C2—C3	−171.9 (5)
Ru3—Ru1—Ru2—C49	−100.27 (16)	C1—C2—C3—C4	−2.0 (10)
C44—Ru1—Ru2—As2	124.6 (11)	C2—C3—C4—C5	−0.1 (10)
C45—Ru1—Ru2—As2	70.34 (17)	C3—C4—C5—C6	1.7 (10)
C46—Ru1—Ru2—As2	−118.13 (15)	C2—C1—C6—C5	−0.8 (8)
As1—Ru1—Ru2—As2	−17.93 (2)	As1—C1—C6—C5	173.3 (4)
Ru3—Ru1—Ru2—As2	167.61 (2)	C4—C5—C6—C1	−1.2 (9)
C44—Ru1—Ru2—Ru3	−43.0 (11)	C1—As1—C7—C12	−71.1 (5)
C45—Ru1—Ru2—Ru3	−97.27 (17)	C13—As1—C7—C12	34.1 (5)
C46—Ru1—Ru2—Ru3	74.26 (15)	Ru1—As1—C7—C12	158.5 (4)
As1—Ru1—Ru2—Ru3	174.47 (2)	C1—As1—C7—C8	107.6 (5)
C26—Sb1—Ru3—C51	4.8 (2)	C13—As1—C7—C8	−147.3 (5)
C38—Sb1—Ru3—C51	122.8 (2)	Ru1—As1—C7—C8	−22.8 (5)
C32—Sb1—Ru3—C51	−112.1 (2)	C12—C7—C8—C9	−1.5 (9)
C26—Sb1—Ru3—C52	−84.0 (2)	As1—C7—C8—C9	179.8 (5)
C38—Sb1—Ru3—C52	34.1 (2)	C7—C8—C9—C10	−0.9 (9)
C32—Sb1—Ru3—C52	159.2 (2)	C8—C9—C10—C11	3.3 (10)
C26—Sb1—Ru3—C50	96.1 (3)	C9—C10—C11—C12	−3.2 (10)
C38—Sb1—Ru3—C50	−145.9 (2)	C8—C7—C12—C11	1.6 (9)
C32—Sb1—Ru3—C50	−20.8 (2)	As1—C7—C12—C11	−179.8 (5)
C26—Sb1—Ru3—Ru2	−162.55 (17)	C10—C11—C12—C7	0.7 (10)
C38—Sb1—Ru3—Ru2	−44.51 (17)	C20—As2—C13—As1	−95.8 (3)
C32—Sb1—Ru3—Ru2	80.56 (16)	C14—As2—C13—As1	159.4 (2)
C26—Sb1—Ru3—Ru1	−179.14 (17)	Ru2—As2—C13—As1	35.6 (3)
C38—Sb1—Ru3—Ru1	−61.10 (16)	C7—As1—C13—As2	80.0 (3)
C32—Sb1—Ru3—Ru1	63.97 (15)	C1—As1—C13—As2	−175.5 (2)
C48—Ru2—Ru3—C51	−16.7 (2)	Ru1—As1—C13—As2	−53.1 (3)
C47—Ru2—Ru3—C51	70.2 (2)	C20—As2—C14—C15	−148.2 (5)
C49—Ru2—Ru3—C51	−110.8 (2)	C13—As2—C14—C15	−42.4 (5)
As2—Ru2—Ru3—C51	145.19 (18)	Ru2—As2—C14—C15	80.2 (5)
Ru1—Ru2—Ru3—C51	173.73 (17)	C20—As2—C14—C19	39.2 (5)
C48—Ru2—Ru3—C52	69.1 (2)	C13—As2—C14—C19	145.1 (4)
C47—Ru2—Ru3—C52	156.0 (2)	Ru2—As2—C14—C19	−92.3 (4)
C49—Ru2—Ru3—C52	−25.0 (2)	C19—C14—C15—C16	−0.9 (9)
As2—Ru2—Ru3—C52	−129.05 (16)	As2—C14—C15—C16	−173.5 (5)
Ru1—Ru2—Ru3—C52	−100.51 (16)	C14—C15—C16—C17	1.4 (11)
C48—Ru2—Ru3—C50	−108.8 (3)	C15—C16—C17—C18	−1.0 (10)
C47—Ru2—Ru3—C50	−21.9 (3)	C16—C17—C18—C19	0.1 (9)
C49—Ru2—Ru3—C50	157.1 (2)	C15—C14—C19—C18	0.0 (8)
As2—Ru2—Ru3—C50	53.08 (19)	As2—C14—C19—C18	172.7 (4)
Ru1—Ru2—Ru3—C50	81.62 (19)	C17—C18—C19—C14	0.4 (9)
C48—Ru2—Ru3—Sb1	150.50 (18)	C14—As2—C20—C25	−124.8 (4)
C47—Ru2—Ru3—Sb1	−122.60 (18)	C13—As2—C20—C25	128.9 (4)
C49—Ru2—Ru3—Sb1	56.36 (16)	Ru2—As2—C20—C25	2.7 (5)
As2—Ru2—Ru3—Sb1	−47.65 (8)	C14—As2—C20—C21	56.2 (5)
Ru1—Ru2—Ru3—Sb1	−19.11 (4)	C13—As2—C20—C21	−50.2 (5)
C48—Ru2—Ru3—Ru1	169.61 (18)	Ru2—As2—C20—C21	−176.3 (4)
C47—Ru2—Ru3—Ru1	−103.49 (17)	C25—C20—C21—C22	−1.3 (8)
C49—Ru2—Ru3—Ru1	75.47 (16)	As2—C20—C21—C22	177.7 (4)
As2—Ru2—Ru3—Ru1	−28.54 (5)	C20—C21—C22—C23	−0.3 (8)
C44—Ru1—Ru3—C51	151.1 (6)	C21—C22—C23—C24	1.6 (8)
C45—Ru1—Ru3—C51	56.9 (6)	C22—C23—C24—C25	−1.2 (8)
C46—Ru1—Ru3—C51	−123.5 (6)	C21—C20—C25—C24	1.7 (8)
As1—Ru1—Ru3—C51	−32.2 (6)	As2—C20—C25—C24	−177.3 (4)
Ru2—Ru1—Ru3—C51	−21.4 (6)	C23—C24—C25—C20	−0.4 (8)
C44—Ru1—Ru3—C52	−110.3 (3)	C38—Sb1—C26—C31	−161.8 (5)
C45—Ru1—Ru3—C52	155.5 (2)	C32—Sb1—C26—C31	95.6 (5)
C46—Ru1—Ru3—C52	−24.8 (2)	Ru3—Sb1—C26—C31	−33.2 (5)
As1—Ru1—Ru3—C52	66.47 (16)	C38—Sb1—C26—C27	23.6 (5)
Ru2—Ru1—Ru3—C52	77.25 (15)	C32—Sb1—C26—C27	−79.0 (5)
C44—Ru1—Ru3—C50	74.9 (3)	Ru3—Sb1—C26—C27	152.2 (4)
C45—Ru1—Ru3—C50	−19.3 (2)	C31—C26—C27—C28	−1.6 (10)
C46—Ru1—Ru3—C50	160.4 (2)	Sb1—C26—C27—C28	173.3 (5)
As1—Ru1—Ru3—C50	−108.33 (17)	C26—C27—C28—C29	0.9 (11)
Ru2—Ru1—Ru3—C50	−97.55 (16)	C27—C28—C29—C30	1.2 (11)
C44—Ru1—Ru3—Sb1	−15.5 (2)	C28—C29—C30—C31	−2.6 (11)
C45—Ru1—Ru3—Sb1	−109.71 (17)	C27—C26—C31—C30	0.1 (9)
C46—Ru1—Ru3—Sb1	69.94 (15)	Sb1—C26—C31—C30	−174.6 (5)
As1—Ru1—Ru3—Sb1	161.25 (4)	C29—C30—C31—C26	1.9 (10)
Ru2—Ru1—Ru3—Sb1	172.030 (19)	C26—Sb1—C32—C33	30.1 (5)
C44—Ru1—Ru3—Ru2	172.5 (2)	C38—Sb1—C32—C33	−69.5 (4)
C45—Ru1—Ru3—Ru2	78.26 (17)	Ru3—Sb1—C32—C33	157.6 (4)
C46—Ru1—Ru3—Ru2	−102.09 (15)	C26—Sb1—C32—C37	−147.0 (4)
As1—Ru1—Ru3—Ru2	−10.78 (4)	C38—Sb1—C32—C37	113.4 (4)
C44—Ru1—As1—C7	106.0 (3)	Ru3—Sb1—C32—C37	−19.6 (5)
C45—Ru1—As1—C7	−160.8 (3)	C37—C32—C33—C34	0.2 (8)
C46—Ru1—As1—C7	14.4 (2)	Sb1—C32—C33—C34	−177.0 (4)
Ru2—Ru1—As1—C7	−80.15 (19)	C32—C33—C34—C35	−1.1 (9)
Ru3—Ru1—As1—C7	−70.92 (19)	C33—C34—C35—C36	2.5 (9)
C44—Ru1—As1—C1	−18.5 (3)	C34—C35—C36—C37	−2.8 (10)
C45—Ru1—As1—C1	74.7 (2)	C35—C36—C37—C32	1.9 (10)
C46—Ru1—As1—C1	−110.1 (2)	C33—C32—C37—C36	−0.5 (8)
Ru2—Ru1—As1—C1	155.33 (17)	Sb1—C32—C37—C36	176.6 (5)
Ru3—Ru1—As1—C1	164.56 (17)	C26—Sb1—C38—C43	70.3 (4)
C44—Ru1—As1—C13	−132.8 (2)	C32—Sb1—C38—C43	168.8 (4)
C45—Ru1—As1—C13	−39.6 (2)	Ru3—Sb1—C38—C43	−57.6 (5)
C46—Ru1—As1—C13	135.6 (2)	C26—Sb1—C38—C39	−108.5 (5)
Ru2—Ru1—As1—C13	41.03 (15)	C32—Sb1—C38—C39	−10.0 (5)
Ru3—Ru1—As1—C13	50.27 (16)	Ru3—Sb1—C38—C39	123.6 (4)
C48—Ru2—As2—C20	−57.7 (2)	C43—C38—C39—C40	0.3 (9)
C47—Ru2—As2—C20	−146.8 (2)	Sb1—C38—C39—C40	179.1 (5)
C49—Ru2—As2—C20	36.0 (2)	C38—C39—C40—C41	1.0 (10)
Ru3—Ru2—As2—C20	140.05 (17)	C39—C40—C41—C42	−1.5 (10)
Ru1—Ru2—As2—C20	115.29 (17)	C40—C41—C42—C43	0.6 (10)
C48—Ru2—As2—C14	63.6 (2)	C39—C38—C43—C42	−1.2 (8)
C47—Ru2—As2—C14	−25.5 (2)	Sb1—C38—C43—C42	−180.0 (4)
C49—Ru2—As2—C14	157.3 (2)	C41—C42—C43—C38	0.7 (9)
Ru3—Ru2—As2—C14	−98.65 (17)		

### Hydrogen-bond geometry (Å, °)

**Table:**

*D*—H···*A*	*D*—H	H···*A*	*D*···*A*	*D*—H···*A*
C13—H13B···O8^i^	0.97	2.59	3.290 (7)	129
C23—H23A···Cg1^ii^	0.93	2.88	3.686 (6)	146
C34—H34A···Cg2^iii^	0.93	2.72	3.564 (6)	151

Symmetry codes: (i) *x*, −*y*+1, *z*−1/2; (ii) *x*, −*y*, *z*−3/2; (iii) −*x*, *y*, −*z*+1/2.
